# Usage and Users of Online Self-Management Programs for Adult Patients With Atopic Dermatitis and Food Allergy: An Explorative Study

**DOI:** 10.2196/resprot.4134

**Published:** 2015-05-23

**Authors:** Harmieke van Os-Medendorp, Ilse van Leent- de Wit, Marjolein de Bruin-Weller, André Knulst

**Affiliations:** ^1^University Medical Centre Utrechtdepartment of Dermatology/AllergologyUtrechtNetherlands; ^2^University Medical Centre Utrechtdepartment of Dermatology/Allergologystudent Clinical Health Sciences; scientific internship department Dermatology/AllergologyUtrechtNetherlands

**Keywords:** self-management, Internet, food allergy, atopic dermatitis

## Abstract

**Background:**

Two online self-management programs for patients with atopic dermatitis (AD) or food allergy (FA) were developed with the aim of helping patients cope with their condition, follow the prescribed treatment regimen, and deal with the consequences of their illness in daily life. Both programs consist of several modules containing information, personal stories by fellow patients, videos, and exercises with feedback. Health care professionals can refer their patients to the programs. However, the use of the program in daily practice is unknown.

**Objective:**

The aim of this study was to explore the use and characteristics of users of the online self-management programs “Living with eczema,” and “Living with food allergy,” and to investigate factors related to the use of the trainings.

**Methods:**

A cross-sectional design was carried out in which the outcome parameters were the number of log-ins by patients, the number of hits on the system’s core features, disease severity, quality of life, and domains of self-management. Descriptive statistics were used to summarize sample characteristics and to describe number of log-ins and hits per module and per functionality. Correlation and regression analyses were used to explore the relation between the number of log-ins and patient characteristics.

**Results:**

Since the start, 299 adult patients have been referred to the online AD program; 173 logged in for at least one occasion. Data from 75 AD patients were available for analyses. Mean number of log-ins was 3.1 (range 1-11). Linear regression with the number of log-ins as dependent variable showed that age and quality of life contributed most to the model, with betas of .35 ( *P*=.002) and .26 (*P*=.05), respectively, and an *R* ^2^ of .23. Two hundred fourteen adult FA patients were referred to the online FA training, 124 logged in for at least one occasion and data from 45 patients were available for analysis. Mean number of log-ins was 3.0 (range 1-11). Linear regression with the number of log-ins as dependent variable revealed that adding the self-management domain “social integration and support” to the model led to an *R* ^2^ of .13. The modules with information about the disease, diagnosis, and treatment were most visited. Most hits were on the information parts of the modules (55-58%), followed by exercises (30-32%).

**Conclusions:**

The online self-management programs “Living with eczema” and “Living with food allergy” were used by patients in addition to the usual face-to-face care. Almost 60% of all referred patients logged in, with an average of three log-ins. All modules seemed to be relevant, but there is room for improvement in the use of the training. Age, quality of life, and lower social integration and support were related to the use of the training, but only part of the variance in use could be explained by these variables.

## Introduction

Atopy refers to the genetic tendency to develop allergic diseases such as atopic dermatitis (AD), allergic rhinitis, asthma or food allergy (FA). Allergic diseases are common in children in the age group up to twelve years; a study showed that at twelve years 58% of the children had AD, asthma, and/or rhinitis at some time [1]. The prevalence of doctor-diagnosed FA is estimated to be 3-8% in children and 1-3% in adults [2]. The prevalence of AD in the Netherlands in children under six years of age is 11.3%, while in adults the prevalence is 2.3% [3]. AD as well as FA has a negative impact on quality of life [4-7].

Technological self-management systems for patients with chronic diseases can help them to understand and monitor their condition, and support patients in achieving behavioral change [8]. Self-management is defined as the individual’s ability to manage symptoms, treatment, physical and psychosocial consequences, and lifestyle changes inherent to living with a chronic condition [9]. Previous studies have shown that interactive eHealth technologies contribute positively to health care for patients with a chronic illness, realizing increased patient-provider communication, positive impact on behavioral change, improved therapy adherence, increased empowerment, and cost reductions [10-12].

We previously developed two online self-management programs based on scientific guidelines and professional experience for patients with AD or FA. The programs were aimed at helping patients cope with their condition, follow the prescribed treatment and deal with the consequences of their illness in daily life. Both programs “Living with eczema” [13], and “Living with food allergy” [14] have a version for adult patients and a version for parents of young children with AD or FA. The modules of the FA program are: (1) What is FA; (2) How is it diagnosed; (3) What to do in case of an allergic reaction; (4) Diet & food allergy; (5) Cross-reactivity; and (6) Coping with FA in daily life. The AD program has the following modules: (1) What is AD; (2) Treatment of AD; (3) Communication; (4) Coping with itch; and (5) Coping with AD in daily life. The programs are in addition to the care of the general practitioner (GP) or medical specialist and were developed in collaboration with patient associations. Both programs are accessible for all Dutch patients after referral to the program by the treating physician, dietician or nurse. Both programs consist of several modules with information, patient experience stories, videos, and exercises with feedback.

In 2010, a feasibility study of the self-management programs for adults took place to explore the usefulness and ease of use of the training. This was based on the Technology Acceptance Model (TAM) developed by Davis [15]. According to this TAM the perceived usefulness and perceived ease of use predict the acceptance and use of technology. The feasibility evaluation showed that both patients and caregivers considered the online training useful and easy to use and they appreciated the content of the training [16]. However, the feasibility study was carried out in a small sample of patients and caregivers and the use of both programs in clinical practice is unknown.

Therefore, the primary objective of this study was to explore the use and characteristics of adult visitors to the online self-management program “Living with eczema” and “Living with food allergy” in order to increase and optimize the use of the program in daily practice. The secondary objective was to investigate the factors related to the use of the program.

## Methods

### Study Design

A cross-sectional research design was used to explore the use of two online self-management programs among patients with AD or FA. The measure of usage was the total number of log-ins in the study period. Data of usage was obtained during the patient’s use of the program and was embedded into the program design. Patients had access to the program for a three month period. The number of log-ins was measured for all participants of both programs, and the number of hits on the system’s core features was measured only among the participants who provided informed consent. To explore the patients’ characteristics a questionnaire-based online survey was conducted on a convenience sample of patients attending the online self-management programs. All patients who provided informed consent were included, and the questionnaires were incorporated at the start of the program.

### Study Participants and Recruitment

Study population consisted of adult patients with FA or with AD who received an account for one of the online programs. To investigate patient characteristics, all patients who provided informed consent since the start of the programs were included. The gender of each patient and the health care provider who enrolled them in the program were registered, and from the start of the program the number of visits to the site was counted. To examine the usage of the program, patients were recruited from the participants of both online self-management programs between October 2012 and November 2013, because since October 2012 it has been possible to measure the number of hits on the system’s core features.

**Figure 1:**
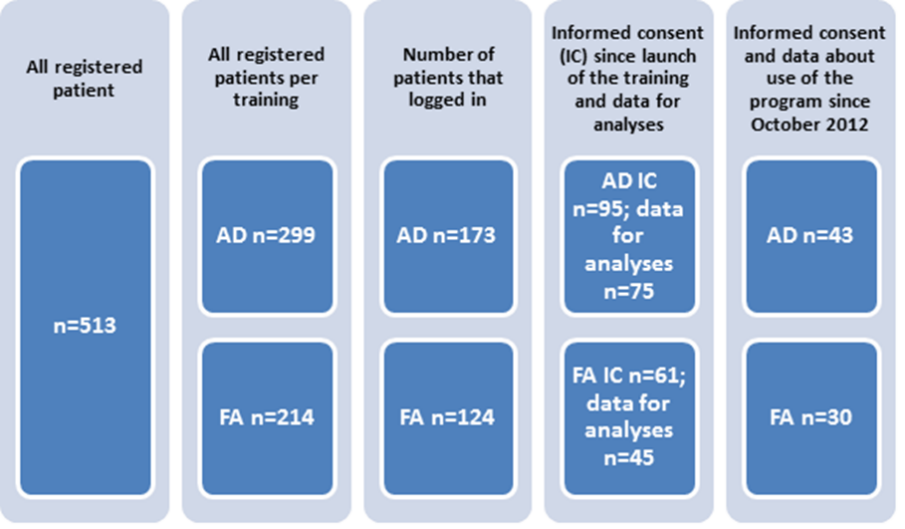
Accounts for the online programs.

Eligible patients were at least 18 years old, Dutch speaking, and had a clinical diagnosis of AD or FA. Patients were referred to the online programs by GPs, specialists, dieticians or nurses. After referral, they received an account from which they were given access to the training. The account was valid for a period of three months in which patients could complete the program, but this period could be extended at the request of the participant. Informed consent was asked at the start of the online program through a specific letter and was incorporated as a link in the webpage of the program. Their reply was registered and they received a copy via email. A flowchart of the study can be found in Figure 1.

The Medical Ethics Review Committee of UMC Utrecht confirmed that the Medical Research Involving Human Subjects Act did not apply to this study.

### Parameters and Research Instruments

#### Demographics

Measurement of the demographic variables (age and gender) was incorporated in the initial questionnaire.

#### Disease Severity

Disease severity of AD was measured using the extent+ severity part of the Impact of Chronic Skin Disease on Daily Life (ISDL) questionnaire [17]. Extent and severity of AD were measured for nine parts of the body: adding up the scores gives the total score of the affected area, ranging from 9 to 36. A Visual Analogue Scale (VAS) ranging from 0 to 100 was used to measure the intensity of itch.

Disease severity characteristics of FA were measured by two questions: (1) which food caused an allergic reaction; and (2) whether the patient had been prescribed an adrenaline auto injector.

#### Quality of Life

Quality of life (QoL) was measured using the Dermatology Life Quality Index (DLQI) for patients with AD. The DLQI is a self-administered general dermatology QoL instrument and consists of ten questions with a 4-point Likert scale ranging from 0 (not at all) to 3 (very much)[18]. The DLQI was translated into Dutch by means of forward-backward translation [19].

QoL of patients with FA was measured using the Food Allergy Quality of Life Questionnaire-Adult Form (FAQLQ-AF). FAQLQ-AF contains 29 items and 4 domains about allergen avoidance & dietary restrictions, emotional impact, risk of accidental exposure, food allergy related health. The total FAQLQ score is the sum of all the items divided by the number of items and ranges from 1 (minimal impairment in health-related quality of life (HRQL)) to 7 (maximal impairment in HRQL) [20].

#### Self-Management

Self-management in patients with both conditions was measured using the health education impact Questionnaire (heiQ) version 3.0 [21]. The heiQ is a self-evaluation instrument that consists of 40 questions on eight different domains: positive and active engagement in life, health directed behavior, skill and technique acquisition, constructive attitudes and approaches, self-monitoring and insight, health service navigation, social integration and support, and emotional well-being. The heiQ items are scored on a Likert scale ranging from 1 (strongly disagree) to 4 (strongly agree). The scoring for the heiQ is a sum-score per subscale, with higher scores indicating higher self-management. The domain emotional well-being is a reverse scale, higher scores mean higher impact on well-being. The heiQ version 3.0 was officially translated into Dutch by means of repeated forward-backward translation and is used in the program.

#### Use of the Program

Number of log-ins and hits per module and per functionality (information, exercises, videos, and patient narratives) were automatically registered in the web system of the online program. This functionality has been available since October 2012.

### Statistical Analyses

Statistical analyses were performed using SPSS Statistics 20.0 (IBM Corporation, Somers, NY, USA). Standard descriptive statistics were used to summarize sample characteristics and to describe numbers of log-ins and hits per module and per functionality.

The total score on the DLQI, FAQLQ-AF and the sum scores on the eight different domains of the heiQ are at interval/ratio level measurements, and correlations with the number of log-ins were calculated using Pearson’s product-moment correlation. The correlation between the extent + severity part of the ISDL, and usage was also calculated using Pearson’s product moment.

For the analysis of factors associated with number of log-ins into the online program, multiple linear regression was used. Variables related to the number of log-ins with a significance level of ≤ 0.1 were included in the model. Categorical variables were converted into dummy variables to perform the regression analysis. Prior to each regression analysis, data were checked for linearity and normality by performing a residual analysis, and checked for multicollinearity.

## Results

### Number of Referrals to the Program

Since the start of both programs, a total of 513 patients received an account for the online program by their physician, nurse or dietician: 299 patients for AD and 214 for FA (Figure 1). Of the AD patients, 62% were women; in the FA program 74% were women. Most patients were referred by the university hospital which developed and started the program (Table 1). The mean number of log-ins for the AD program was 1.4 (range 0-15). For the FA program the mean number of log-ins was 1.3 (range 0-16); 58% of patients of both programs logged in on at least one occasion.

**Table 1:** Referral to the online program.

Referral to the online program	Food allergy n(%)	Atopic dermatitis n(%)
University hospital	196 (92%)	178 (60%)
Dietician	3 (1%)	-
General hospital	12 (6%)	108 (36%)
General practitioner	3 (1%)	11 (4%)
Other		2 (1%)
Total	214	299

### Users’ Characteristics of the Online Program “Living With Eczema”

Of the 299 AD patients referred to the online program, 173 logged in on at least one occasion and 95 gave informed consent for the use of their data. Eighty-one patients filled in questionnaires, but 6 were excluded because they were under 18 years of age (n=4) or age was unknown (n=2). Reasons for not using the program or no informed consent were not given, because patients were asked to give their informed consent (yes or no) online when first visiting the program.

Data of 75 patients was available for analysis (Table 2). Of them 67 % were women and the mean age was 34 years (SD 15). Mean number of log-ins was 3.1 (range 1-11; SD 2.6). Patients had mild to moderate AD; mean ISDL 18.8 (range 10-34) and a mean VAS score of itching of 6.0 (range 1-10). AD had a moderate effect on patients’ lives (mean score DLQI 9.6; range 0-27).

A low, but significant correlation was shown between the number of log-ins and age (Pearson correlation coefficient r=.38, *P*=.001), and between number of log-ins and quality of life (r=.32, *P* ˂.01). Correlations between number of log-ins and domains of self-management, such as emotional well-being and skills and techniques were .2 (*P=*.09) and -.19 (*P=*.1), respectively. No correlation was shown between number of log-ins and sex or with other domains of self-management. Linear regression with number of log-ins as dependent variable and entering age, quality of life, and two domains of self-management, namely emotional well-being and skills and techniques, led to an *R* ^2^of .23. Age and quality of life contributed the most to the model, with betas of .35 (*P=*.002) and .26 (*P*=.05), respectively. Two of the self-management domains, emotional well-being, and skills and techniques did not significantly contribute to the model.

**Table 2:** Characteristics of users of the online program “Living with eczema”.

Characteristics		Mean (SD) (range)
Age in years		34.4 (14.8) (18-78)
Number of log-ins		3.1 (2.6) (1-11)
Severity of AD (ISDL score)		18.8 (4.9) (10-34)
Intensity of itching (VAS)		6.0 (2.7) (1-10)
Quality of life		9.6 (7.0) (0-27)
**Domains of self-management**		
	Health directed behavior	11.4 (2.4) (4-16)
	Positive and active engagement in life	15.0 (2.8) (5-20)
	Emotional well-being	12.4 (3.7) (6-22)
	Self-monitoring and insight	16.7 (2.2) (6-22)
	Constructive attitude and appeal	15.5 (2.9) (5-20)
	Skills and techniques	10.3 (2.1) (4-16)
	Social integration and support	14.6 (2.9) (5-20)
	Health service navigation	14.8 (2.4) (5-20)

### Users’ Characteristics of the Online Program “Living With Food Allergy”

Of the 214 FA patients referred to the online program, 124 logged in at least once and 61 gave informed consent for the use of their data. Forty-nine patients filled in questionnaires, but 4 were excluded because they were under 18 years of age. Reasons for not using the program or no informed consent were not given.

Data of 45 patients were available for analysis (Table 3). Of them 80 % were women and mean age was 35 years (SD 13). Mean number of log-ins was 3.0 (range 1-11; SD 2.3). The mean number of food allergies was 3.5 (range 1-8), with highest percentages of allergies for tree nuts, peanut, and fruit/vegetables. Of them, 73% had been prescribed an adrenalin pen.

A low, but significant negative correlation was shown between the number of log-ins and the domain of self-management “social integration and support” (r=-.36, *P*=.02). No correlation was shown between number of log-ins and sex, age, possession of adrenaline pen, aspects of quality of life, and other domains of self-management. Linear regression with number of log-ins as dependent variable and entering “social integration and support” led to an *R* ^2^of .13.

**Table 3:** Characteristics of users of the online program “Living with food allergy”.

Characteristics		Score
Gender (female), n (%)		36 (80%)
Age, mean in years (SD)(range)		34.6 (12.5) (18-64)
**Type of food allergy, n (%)**		
	Peanut	27 (60%)
	Tree nuts	36 (80%)
	Vegetables + fruits	29 (64%)
	Milk	11 (24%)
	Egg	8 (18%)
	Seafood	5 (11%)
	Sesame	7 (16%)
	Other	3 (7%)
Number of food allergies,^,^mean (SD) (range)		3.5 (1.9)(1-8)
In possession of EpiPen, n (%)		33 (73.3%)
Number of log-ins, mean (SD) (range)		3 (2.3)(1-11)
**Domains of self-management mean (SD) (range)**		
	Health directed behavior	11.9 (2.6)(7-16)
	Positive and active engagement in life	16.1 (2. 5)(10-20)
	Emotional well-being	11.0 (3.7)(6-17)
	Self-monitoring and insight	17.1 (2.2)(10-21)
	Constructive attitude and appeal	16.6 (2.6)(10-20)
	Skills and techniques	11.2 (1.9) (6-16)
	Social integration and support	15.3 (2.4) (10-20)
	Health service navigation	15.3 (2.2) (9-20)
**Food allergy quality of life mean (SD) (range)**		
	Allergy avoidance & dietary restrictions	3.5 (1.2) (1.2-6.0)
	Emotional impact	3.9 (1.4) (1.0-6.1)
	Risk of accidental exposure	3.9 (1.2) (1.1-5.8)
	Food allergy related health	3.8 (1.7) (1.3-7.0)
	Food allergy quality of life total score	3.8 (1.1) (1.2-5.5)

### Use of the Different Modules of the Program

The modules “What is AD” and “Treatment of AD” were the most visited modules of the AD program with 34% and 32% of all hits respectively. The module “How is it diagnosed” in the FA program was most visited with 24% of all hits (Table 4).

Most hits (excluding hits on introduction of a module) were on the informational parts of the modules (55-58%), followed by exercises (30-32%). Ten percent or less of all hits were on videos and patient narratives (Table 5).

**Table 4:** Use of the different modules of the program.

Atopic dermatitis (n=43 patients ; total of 109 log-ins)		Food allergy (n=30 patients; total of 65 log-ins)	
Usage per module	Number of hits (%)	Usage per module	Number of hits (%)
What is AD	179 (34%)	What is FA	127 (18%)
Treatment of AD	171 (32%)	How is it diagnosed	172 (24%)
Communication	46 (9%)	What to do in case of allergic reaction	120 (17%)
Coping with itch	78 (15%)	Diet & food allergy	125 (17%)
Coping with AD in daily life	56 (11%)	Cross-reactivity	53 (7%)
		Coping with FA in daily life	128 (18%)
Total number of hits	530	Total number of hits	725

**Table 5:** Use of different functionalities per program.

Number of hits	Living with eczema (n=43 patients)	Living with food allergy^a^ (n=30 patients)
Informational parts	307 (58%)	331(55%)
Exercises	157 (30%)	189 (32%)
Videos	33 (6%)	57 (10%)
Patient narratives	33 (6%)	21 (4%)

^a^Hits on the introduction of a module were not counted.

## Discussion

### Principal Findings

The online self-management programs “Living with eczema” and “Living with food allergy” were mostly used by patients of our university center. Of all referred patients, 58% logged in for at least one occasion. Patients who participated in the online program had an average of three log-ins and mostly visited the modules with information about the disease (AD or FA) and its treatment. Most hits were on the informational sections of the modules and on exercises. It seemed that higher age and lower quality of life influenced use of the AD program, while the lower scores on “social integration and support” influenced use of the FA program. However, explained variance of use was low.

The feasibility evaluations of both online programs, carried out previously, showed that usefulness and usability of the programs were well-appreciated. According to the TAM model [15], this could predict use of the program. However, the underlying study showed that about 40% of patients referred to the online program never logged in. Because no data are available about patients who received an account but chose not to use the program, we do not know if there were differences between users and non-users. It is known that high attrition rates in eHealth interventions are not uncommon [8,22,23]. We did not investigate reasons for the non-usage attrition, but some patients mentioned during face-to-face consultations at the outpatient department that they had already received enough information. So it could be that the “right” users, who could benefit the most from the program, were not enrolled in all cases, which might lead to increased non-use [22,24]. Moreover, the online program is not fully integrated in the usual clinical face-to-face care, but was offered as an addition to usual care, which could also lead to non-use [23]. During the program, there was little room for human interaction. Patients could only receive feedback from a nurse on one specific exercise in the AD program. In the FA program, patients received automatic feedback on most exercises and they received personal feedback from a dietician on only one exercise. It was earlier reported that involvement of interactive technologies with human interaction and support can reduce attrition rates [8,24,25].

Besides the factors related to the online program itself, patient characteristics may also influence the use of the program. We concluded that higher age and lower quality of life were associated with use of the “Living with AD” program, but the explained variance was 23%. The mean age of users of the AD program was 34 years. It is possible that relatively older patients more often took the opportunity to visit the program than young adults. Usage of eHealth interventions has also been studied in patients with other chronic diseases. For example, users of a health weight assistant [26] and a Web-based intervention for heart disease self-management [27] were also more often of older age. Besides age, it was pointed out that the need for information or the need for care influenced use of the eHealth intervention [24,27], which was confirmed by our finding that decreased quality of life increased use of the online AD program. The FA patients in this study had a moderately impaired quality of life (mean score 3.8, SD 1.1). However, quality of life was not correlated to the use of the online program as in the AD patients. One explanation might be that for both patient groups, disease specific quality of life questionnaires were used, which were not comparable due to different aspects of quality of life being measured. On the other hand, lower scores on “social integration and support”, a domain of self-management, influenced the number of log-ins by FA patients. It is known that the social consequences of having a food allergy, such as feeling isolated or embarrassed, also influence quality of life [6].

Because of the low explained variance in the use of both programs, other factors than demographics, disease severity, quality of life, and self-management may be of influence. We expect that eHealth literacy is such a factor. eHealth literacy is defined as “*the ability to seek, find, understand, and appraise health information from electronic sources and apply the knowledge gained to addressing or solving a health problem*” [28]. eHealth literacy is not a static skill but develops over time and can be influenced by the health status of a person, his educational background, motivation to look for information, and the technologies that are used [28]. The eHealth literacy of participants of this study are not known, but it is of interest to further explore these skills and investigate their relation to the use of these online programs.

There is no doubt about the importance of patient education and self-management support for patients with AD [29] or FA [30]. However, little is known about the most preferred method for these patients. In the studied online programs, the information parts were most frequently visited, while we expected that videos and patient narratives would be more appealing. It was shown in one earlier study that online videos providing patient education were effective and attractive to patients with AD [31]. Another Web-based program for AD patients also found lower use of videos than expected, despite careful development of that program and adaptation after initial feedback [32]. Parents of food allergic children reported that they preferred a variety of formats for patient education, because of the differences in learning styles: paper-based, Web-based, and video-based information. They also preferred that these materials were recommended by reliable organizations [33,34]. We expected that the high use of the informational parts of these programs might also be related to the low number of log-ins. The different modules of the AD and FA program always start with the information pages, and participants have to take further steps for the online exercises and videos. A change in the order in which the different functionalities are offered might give the users more freedom of choice in using the online program.

### Limitations

A limitation of the study was that disease severity, quality of life, and self-management were only measured at the start of the program; as a result, effects of the program on these clinical outcomes are unknown and it is not possible to analyze which patients will benefit most from the program. Further research with a longitudinal design will give additional insight into the effects of the programs. Moreover, most patients were referred by a university center. In this center specialized nurses or dieticians already support patients with information and education The actual need for information in this university center may be lower in than in less specialized or general hospitals. Implementation of the program at regional hospitals or in community care would probably increase the use of the programs and extend it to a more diverse patient group. Knowledge about use and factors influencing use of specific self-management programs can contribute to optimal usage of these programs, which in turn will increase the intended effects on clinical outcomes. However, generalizability of the findings of this study is limited due to the small sample size and specific adult patient group.

### Conclusion

Physicians, dieticians, and nurses, mostly from a university center, regularly referred their patients to the online programs “Living with food allergy” or “Living with eczema” for online self-management training, in addition to the usual face-to-face care. Nearly 60% of all referred patients logged in. All modules seemed to be relevant, but there is room for improvement in use of the program. Age, quality of life, and lower social integration and support were related to use of the program, but only a part of variance in use could be explained. Further research is needed into predictors of use related to the program and characteristics of users, as well as further research into the effects of the program on clinical outcomes.

## Abbreviations

AD: atopic dermatitis
DLQI: Dermatology Life Quality Index
FA: food allergy
FAQLQ-AF: food allergy quality of life questionnaire-adult Form
heiQ: health education impact questionnaire
HRQL: health-related quality of life
ISDL: Impact of Chronic Skin Disease on Daily Life
QoL: quality of life
VAS: Visual Analogue Scale
